# Distinct mechanisms involving diacylglycerol, ceramides, and inflammation underlie insulin resistance in oxidative and glycolytic muscles from high fat-fed rats

**DOI:** 10.1038/s41598-021-98819-7

**Published:** 2021-09-27

**Authors:** Shailee Jani, Daniel Da Eira, Ishvinder Hadday, George Bikopoulos, Arta Mohasses, Ricardo A. de Pinho, Rolando B. Ceddia

**Affiliations:** 1 Muscle Health Research Center – School of Kinesiology and Health Science, York University, 4700 Keele St, North York, ON M3J 1P3 Canada; 2 Graduate Program in Health Sciences, School of Medicine, Pontifícia Universidade Católica Do Paraná, Curitiba, Paraná, Brazil

**Keywords:** Biochemistry, Physiology, Diseases, Endocrinology

## Abstract

This study investigated whether oxidative and glycolytic rat skeletal muscles respond differently to a high-fat (HF) sucrose-enriched diet with respect to diacylglycerol (DAG) and ceramides accumulation, protein kinase C (PKC) activation, glucose metabolism, and the expression of inflammatory genes. HF diet (8 weeks) suppressed insulin-stimulated glycogen synthesis and glucose oxidation in soleus (Sol), extensor digitorum longus (EDL) and epitrochlearis (Epit) muscles. However, DAG and ceramides levels increased in Sol and EDL, but not in Epit muscles of HF-fed rats. Additionally, membrane-bound PKC-delta and PKC-theta increased in Sol and EDL, whereas in Epit muscles both PKC isoforms were reduced by HF diet. In Epit muscles, HF diet also increased the expression of tumor necrosis factor-α (TNF-α) receptors (CD40 and FAS), toll-like receptor 4 (TLR4), and nuclear factor kappa light polypeptide gene enhancer in B cells (NF-kB), whereas in Sol and EDL muscles the expression of these inflammatory genes remained unchanged upon HF feeding. In conclusion, HF diet caused DAG and ceramides accumulation, PKC activation, and the induction of inflammatory pathways in a fiber type-specific manner. These findings help explain why oxidative and glycolytic muscles similarly develop insulin resistance, despite major differences in their metabolic characteristics and responsiveness to dietary lipid abundance.

## Introduction

Besides encompassing a large proportion of total body mass^1^, skeletal muscles have the capacity to store up to 1 to 2% of their weight in glycogen^2^, accounting for the majority of whole-body glucose uptake and virtually the entire non-oxidative glucose metabolism^3^. In type 2 diabetes (T2D) patients, the insulin-mediated glucose disposal is reduced to about 50% of the values of non-diabetic control subjects. Such reduction in glucose disposal is mainly due to impaired skeletal muscle glycogen synthesis^4^. Therefore, proper regulation of whole-body glucose homeostasis is dependent on the preserved ability of skeletal muscles to synthesize and store glycogen in response to insulin.

Several mechanisms have been proposed to explain the pathogenesis of obesity-induced insulin resistance in skeletal muscles. These include excessive intracellular accumulation of lipids^5–7^, mitochondrial dysfunction and reduced oxidative capacity^8–10^, elevated levels of inflammatory cytokines^5,11^, and increased oxidative stress^12,13^. Although all of these have been associated with impaired insulin signalling and glucose metabolism, no single mechanism seems to explain how skeletal muscles with different metabolic characteristics develop insulin resistance under conditions of obesity^14^. From the perspective of lipotoxicity, the accumulation of lipid intermediates like DAG and ceramides and the activation of PKC have been implicated in insulin resistance by triggering pathways that lead to serine phosphorylation of IRS-1, thereby suppressing insulin-stimulated glucose metabolism^5,7^. Also, the activation of proinflammatory pathways by elevated production of reactive oxygen species (ROS) has been proposed as a mechanism by which increased oxidation of non-esterified fatty acids (NEFAs) leads to insulin resistance in skeletal muscles^11,12^. However, it has been reported that PKC content and activation, as well as ROS production associated with NEFA oxidation in rat skeletal muscles display fiber type-specific patterns^15,16^. In fact, the amount of PKCθ was 2.5 times higher in the white tensor fascia latae muscle compared with the red Sol muscle, with the mixed muscles vastus intermedius and plantaris having intermediate levels of this kinase^16^. It has also been reported that H_2_O_2_ emission rates in red Sol were much lower than in the mixed EDL and the white Epit muscles^15^. In this context, it is plausible that the mechanisms governing diet-induced insulin resistance vary depending on the fiber type composition of various skeletal muscles. This is particularly relevant if one considers that in diet-induced obesity, insulin resistance develops in all muscles, despite their variability in fiber type distribution. Thus, it may be that, under obesogenic conditions, DAG-mediated PKC activation leads to impaired insulin signalling and glucose metabolism in oxidative muscles, whereas the presence of elevated circulating inflammatory cytokines and the ability to respond to them and to other endocrine/metabolic factors could be the main determinants of insulin resistance in glycolytic muscles.

Obesity is indeed characterized by a state of low-grade inflammation in which TNF-α, interleukin-1β (IL-1β), and interleukin-6 (IL-6)^11^ are chronically elevated. Furthermore, insulin resistance has been associated with hyperactivity of the hypothalamic-pituitary adrenal axis and excess circulating glucocorticoids in animal models of obesity^17–19^, whereas treatment of rats with an anti-glucocorticoid drug ameliorated HF diet-induced skeletal muscle insulin resistance^20^. These observations provide support to the idea that circulating factors (e.g*.,* inflammatory cytokines and hormones) and altered intramuscular lipid metabolism, either independently or in combination, determine the pathophysiology of obesity-induced skeletal muscle insulin resistance. To test this hypothesis, we measured insulin-stimulated glycogen synthesis and glucose oxidation, DAG and ceramides content, membrane-associated PKCδ and PKCθ levels, and the expression of inflammatory genes in slow- and fast-twitch skeletal muscles extracted from rats exposed for 8 weeks to a HF diet. This study provides evidence that the accumulation of DAG and ceramides, PKCδ and PKCθ activation, as well as the expression of inflammatory genes and glucocorticoid receptor (GR) differ significantly between highly oxidative and highly glycolytic skeletal muscles under conditions of HF diet-induced obesity. The findings support that HF feeding causes impairment of glucose metabolism in predominately slow- or fast-twitch rat muscles through distinct mechanisms.

## Results

### Body weight, NEFAs, IL-6, TNF-α, and corticosterone

**Figure 1:**
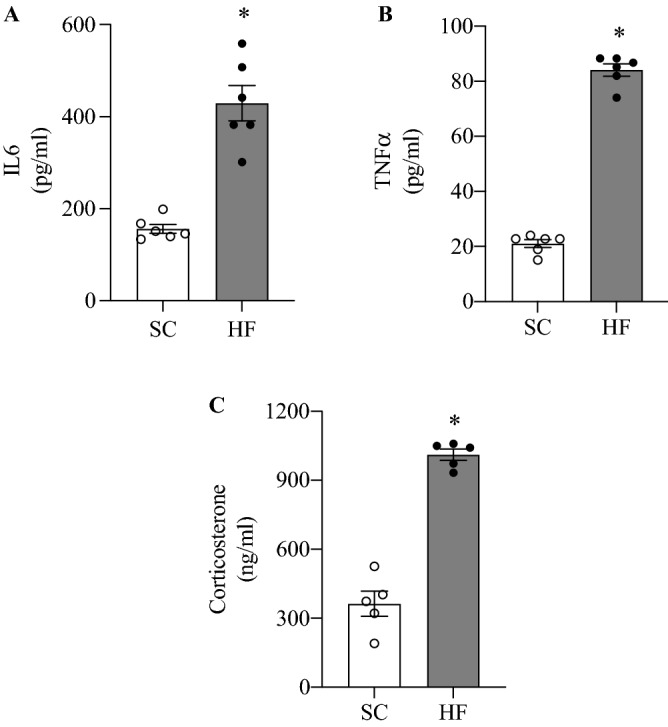
Circulating IL-6 (**A**), TNF-alpha (**B**), and corticosterone (**C)** are increased after 8 weeks of HF feeding in comparison SC feeding. *p < 0.05 vs. SC, *t*-test, n = 5–6.

Both groups of animals progressively increased body weight during the study-period, although the rate of weight gain was significantly higher in HF-fed than control rats. In fact, HF-fed rats weighed 6.6% and 8.2% more than controls after 4 and 8 weeks of diet intervention, respectively (Table 1). Time-course analysis of circulating NEFAs in the fed state revealed that this parameter did not differ between control and HF rats (Table 1). At week 8 of the dietary intervention, HF-fed rats also had circulating IL-6, TNF-α, and corticosterone elevated by 2.75-fold (Fig. 1A), 4-fold (Fig. 1B), and 2.8-fold (Fig. 1C), respectively, when compared to control SC-fed rats.

**Table 1:** Time-course alterations in body weight and circulating NEFAs in Con and HF-fed rats.

		Duration of the study (Weeks)			
		0	2	4	8
Body weight (g)	Con	231.89 ± 2.98	290.63 ± 4.02	383.26 ± 5.87	501.80 ± 11.46
	HF	233.95 ± 3.84	298.21 ± 4.51	408.37 ± 5.94*	543.08 ± 7.43^#^
NEFAs (mM)	Con	0.343 ± 0.037	0.309 ± 0.017	0.376 ± 0.028	0.414 ± 0.052
	HF	0.372 ± 0.032	0.373 ± 0.025	0.418 ± 0.036	0.483 ± 0.041

*P < 0.05 vs. Con 4 weeks; ^#^p < 0.05 vs. Con 8 weeks. n = 10.

### Glycogen synthesis and glucose oxidation in Sol, EDL, and Epit muscles

**Figure 2:**
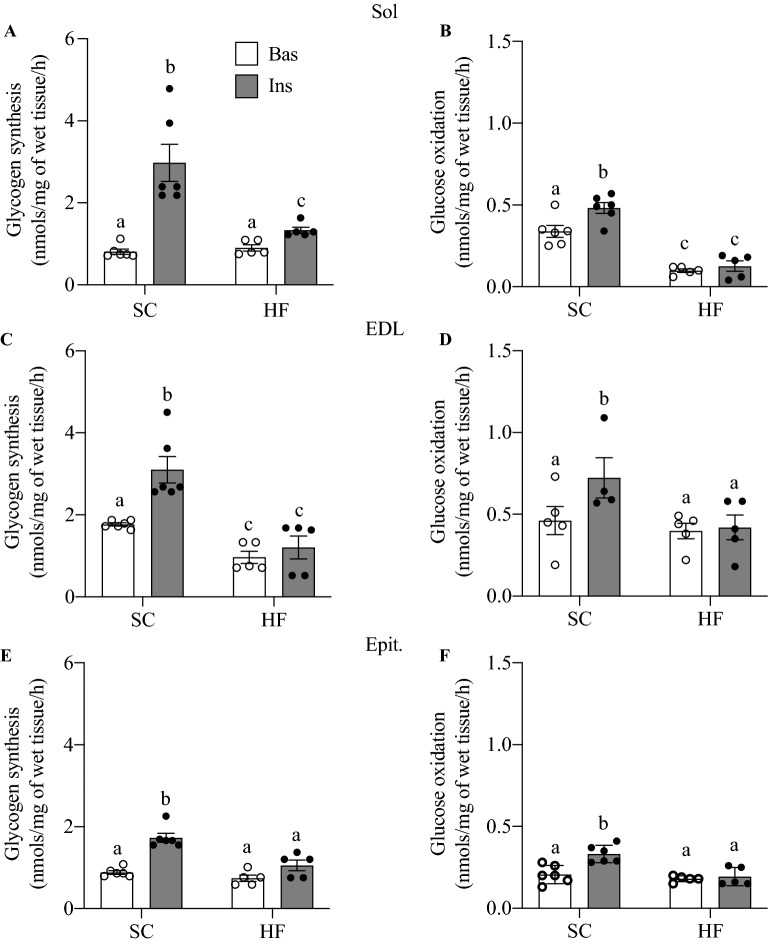
HF diet inhibits insulin-stimulated glycogen synthesis (**A**,**C**,**E**) and glucose oxidation (**B**,**D**,**F**) in Sol, EDL, Epit rat muscles. Distinct letters denote statistical significance (p < 0.05), Two-way ANOVA, n = 5–6.

Glycogen synthesis was similar in Sol and Epit muscles of control and HF-fed rats under basal conditions. In contrast, EDL muscles from HF rats showed lower rates of glycogen synthesis under basal conditions when compared to controls. As expected, insulin-stimulated rates of glycogen synthesis increased by 3.68-fold in Sol (Fig. 2A), 1.75-fold in EDL (Fig. 2B), and 1.97-fold in Epit (Fig. 2C) muscles of control rats; however, this variable was significantly reduced in all three muscles of HF rats. In the presence of insulin, glucose oxidation was also increased by 1.41-fold in Sol (Fig. 2D), 1.57-fold in EDL (Fig. 2E), and 1.57-fold in Epit (Fig. 2F) muscles of SC rats, whereas in all three muscles from HF rats, insulin-stimulated glucose oxidation was potently suppressed. These findings indicate that Sol, EDL, and Epit muscles similarly developed impaired insulin-stimulated glucose metabolism upon HF feeding.

### RER and palmitate oxidation

**Figure 3:**
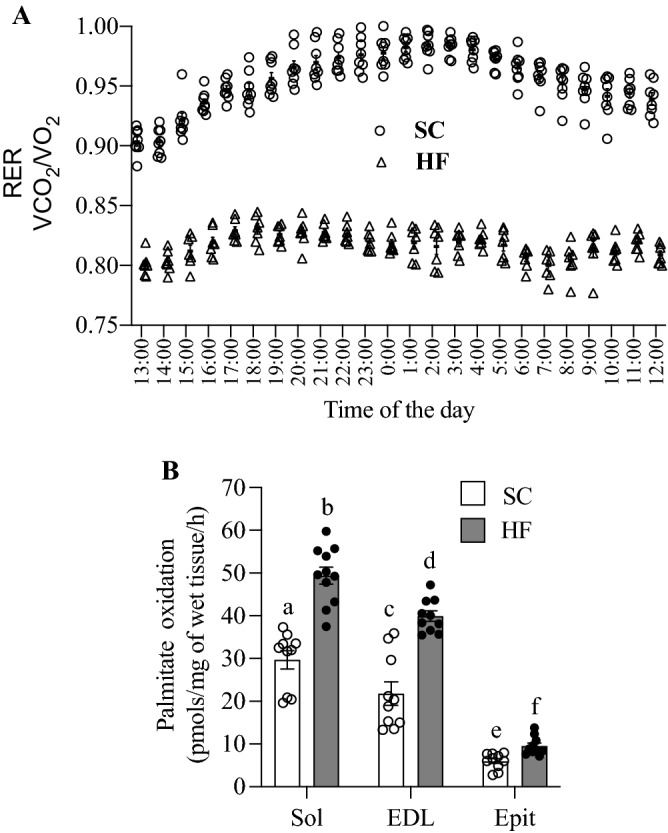
HF diet increases whole-body (**A**) and skeletal muscle (**B**) fat oxidation. Strips of Sol, EDL, and Epit muscles from SC and HF-fed rats were assayed for ^14^CO_2_ production from ^14^C-palmitic acid at week 8 of the study. Twenty four-hour RER was measured at week 8 of the study. Palmitate oxidation and RER are presented as average ± SEM. Distinct letters denote statistical significance (p < 0.05), Two-way ANOVA, n = 8–11.

RER averaged 0.955 ± 0.003 and 0.824 ± 0.009 for control and HF rats, respectively (Fig. 3A), indicating that the latter significantly increased whole-body fat oxidation. Also, as expected, rates of palmitate oxidation in SC-fed rats were much higher in soleus and EDL than Epit muscles (Fig. 3B). In HF-fed rats, rates of palmitate oxidation followed a similar pattern and significantly increased in all muscles, reaching values 1.66-, 1.83-, and 1.59-fold higher than Sol, EDL, and Epit muscles from SC-fed rats, respectively (Fig. 3B). Thus, our findings indicate that energy substrate partitioning was shifted towards fatty acid oxidation in HF-fed rats.

### DAG and ceramides contents in Sol, EDL, and Epit muscles

**Figure 4:**
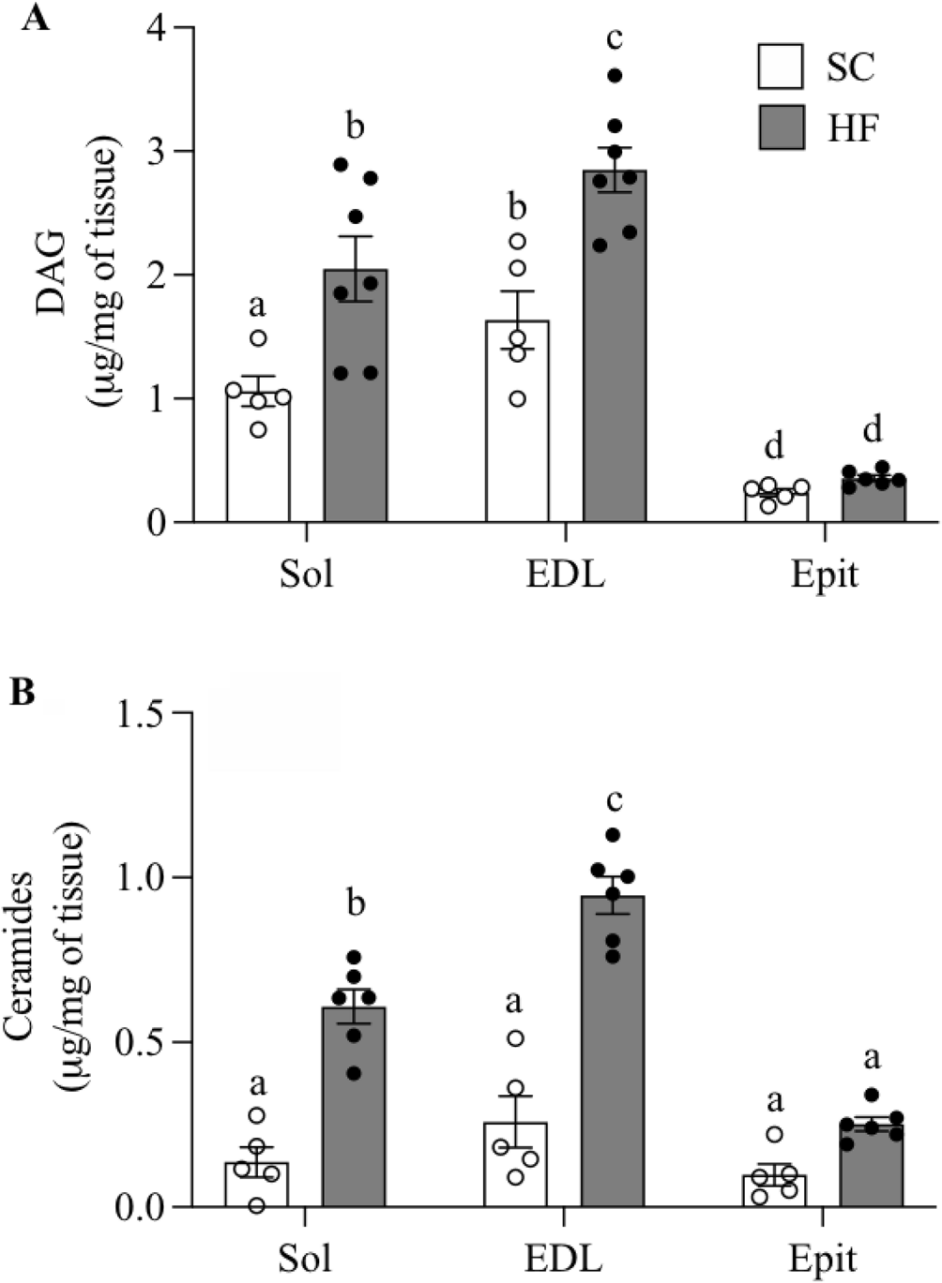
DAG (**A**) and ceramides (**B**) levels increase in Sol and EDL, but not in Epit rat muscles after 8 weeks of feeding a HF diet. Distinct letters denote statistical significance (p < 0.05), Two-way ANOVA, n = 5–6.

In SC-fed rats, DAG content was highest in EDL (1.63 ± 0.23 mg/mg of tissue) followed by Sol (1.06 ± 0.12 mg/mg of tissue) and Epit (0.24 ± 0.03 mg/mg of tissue) muscles (Fig. 4A). HF feeding significantly increased DAG content by 1.74- and 1.93-fold in EDL and Sol, respectively, whereas in Epit muscles this variable did not significantly differ between SC and HF animals (Fig. 4A). In SC-fed rats, ceramides content was also highest in EDL (0.26 ± 0.08 mg/mg of tissue), although no significant differences were found between Sol (0.14 ± 0.05 mg/mg of tissue) and Epit (0.10 ± 0.03 mg/mg of tissue) muscles (Fig. 4B). Upon HF feeding, ceramides content in EDL and Sol muscles significantly increased by 3.66- and 4.46-fold, respectively. In Epit muscles from HF-fed rats, ceramides content was 2.67-fold higher than in Epit muscles from SC-fed rats, however, it did not reach statistical significance (Fig. 4B). These findings indicate that muscles displaying higher rates of fatty acid oxidation not only were richer in DAG and ceramides, but also accumulated higher amounts of these intermediary lipids upon HF feeding.

### PKCδ and PKCθ content and localization in Sol, EDL, and Epit muscles

**Figure 5:**
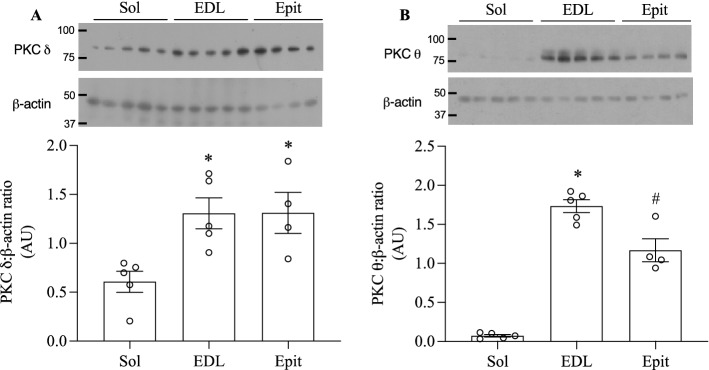
Higher levels of PKCδ (**A**) and PKCθ (**B**) in EDL and Epit than in Sol muscles. Densitometric analyses show arbitrary units (AU) for PKCδ and PKCθ levels divided by β-actin. *p < 0.05 vs. Sol, ^#^p < 0.05 vs. Sol and EDL, One-way ANOVA, n = 4–5.

**Figure 6:**
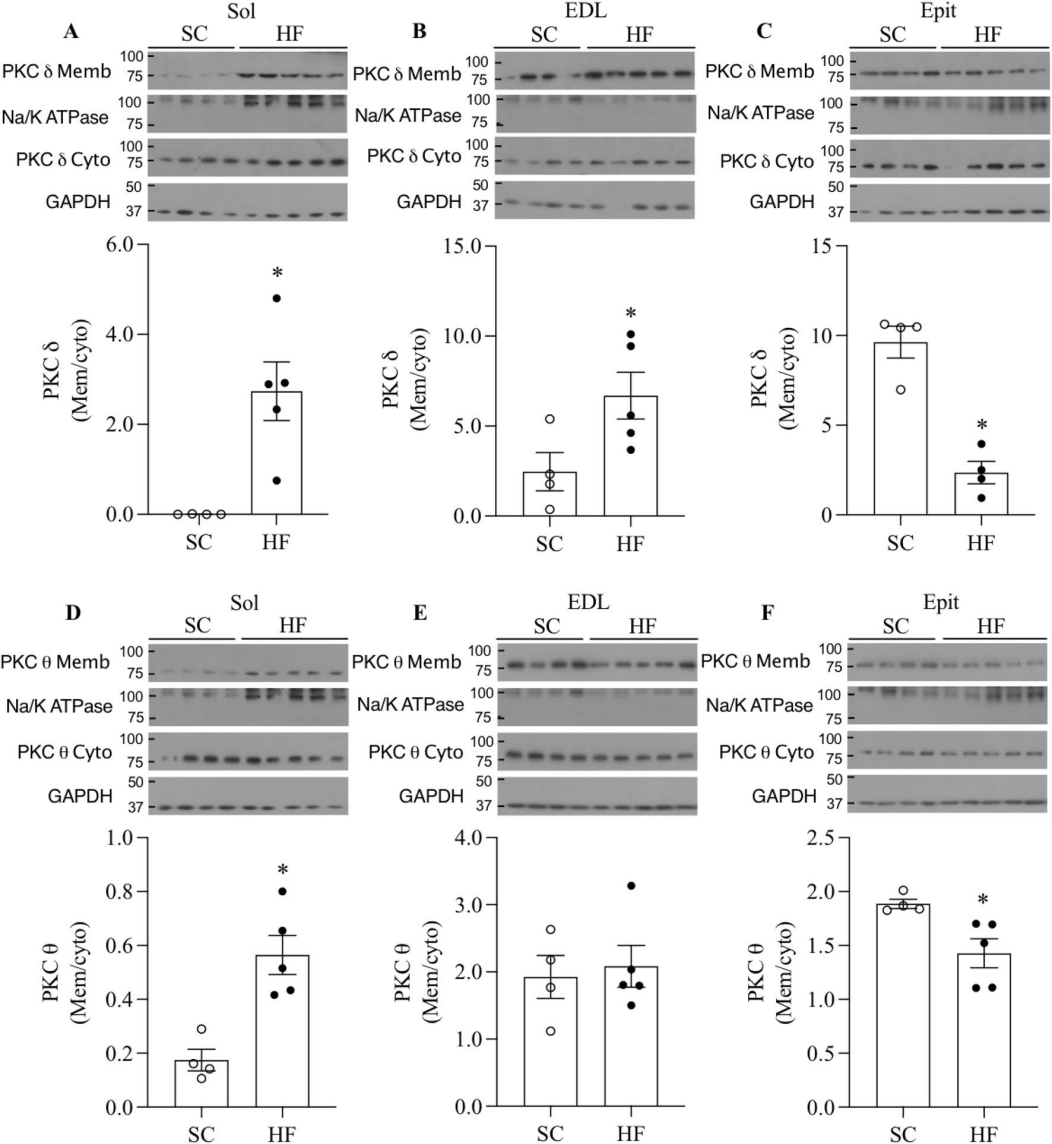
Distinct effects HF diet on PKC translocation towards the membrane in Sol, EDL, and Epit muscles. Representative blots and densitometric analyses of PKCδ, Na/K ATPase, and GAPDH (**A**–**C**) and PKCθ, Na/K ATPase, and GAPDH (**D**–**F**) levels in membrane (Memb) and cytoplasmic (Cyto) cellular fractions. Densitometric analyses show PKCδ and θ ratios (mem/cyto) as an index of activation. *p < 0.05, *t*-test, n = 4–5.

PKCθ is recognized as the dominant isoform of DAG-sensing PKCs in skeletal muscles^21^. However, PKCδ displays the highest sequence similarity (67%) to PKCθ^22^ and it has been shown to translocate to the membrane^23^ and regulate insulin sensitivity and skeletal muscle metabolism^24^. Thus, in this study we measured levels of these two PKC isoforms in Sol, EDL, and Epit muscles. We found that PKCδ levels were similar between EDL and Epit, whereas in Sol its levels were approximately half of those of EDL and Epit muscles (Fig. 5A). These differences were even bigger for PKCθ, with its levels in Sol muscles being only 4% and 6% of those of EDL and Epit muscles, respectively (Fig. 5B). Translocation of PKCδ and PKCθ from cytosol to plasma membrane, reflecting PKC activation, was determined by measuring the membrane/cytoplasm ratios of both PKC isoforms. First, the densitometric values of the PKC membrane and cytoplasm blots were normalized by the densitometric values of Na/K ATPase and GAPDH, respectively. The normalized values for the membrane fractions were then divided by their respective normalized cytoplasm fractions. In HF-fed Sol and EDL muscles, the membrane/cytoplasm ratios for PKCδ were significantly increased by 26.9- and 2.77-fold, respectively (Fig. 6A,B), whereas for PKCθ, only the HF-fed Sol muscle had the membrane/cytoplasm ratio significantly elevated (1.68-fold) in comparison to SC-fed muscles (Fig. 6D,E). Conversely, Epit muscles from HF-fed rats displayed 34% and 24% lower membrane/cytoplasm ratios than SC muscles for PKCδ and PKCθ, respectively (Fig. 6C,F). These findings indicate that HF diet increased PKC activity in Sol and EDL muscles and reduced the activity of this kinase in Epit muscles.

### mRNA expression of inflammatory mediators, glucocorticoid receptor (GR), and 11β-hydrohysteroid dehydrogenase type 1 (11β-HSD1)

**Figure 7:**
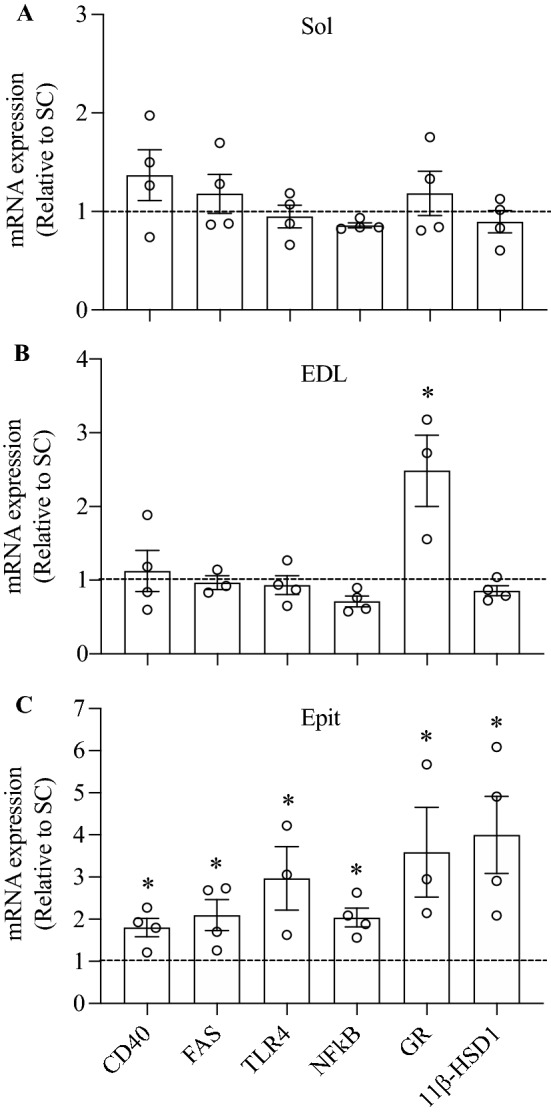
The expression of receptors for inflammatory mediators and glucocorticoids is up-regulated in a fiber type-specific manner by HF diet. mRNA expression of TNF receptors (CD40 and FAS), TLR4, NF-kB, GR, and 11β-HSD1 was measured in Sol, EDL, and Epit muscles. Data presented relative to SC-fed rats (dashed lines). Average ± SEM. *p < 0.05 vs. control, *t*-test, n = 3–4.

Quantitative PCR analysis revealed that the mRNA levels of CD40 and FAS, TLR4, and NF-kB were not altered in Sol and EDL muscles of HF rats (Fig. 7A,B). Only the mRNA expression of the GR was significantly increased (~ 2.5-fold) in EDL muscles of HF rats when compared to SC-fed controls (Fig. 7B). However, in Epit muscles form HF rats, the mRNA expression of CD40, FAS, TLR, GR, and 11β-HSD1 significantly increased by 1.8-fold, 2.1-fold, 2.6-fold, 2.0-fold, 3.6-fold, and 3.7-fold, respectively (Fig. 7C). Thus, a clear distinct fiber-type pattern of expression for inflammatory markers and GR was detected upon HF feeding.

## Discussion

Here, we provide evidence of a muscle fiber type-specific adaptation with regards to the accumulation of DAG and ceramides, PKC activation, and the expression of receptors involved in inflammatory cytokines and glucocorticoid signalling in response to chronic exposure to a HF-sucrose enriched diet. Insulin is well known for its ability to potently stimulate glucose uptake and glycogen synthesis in skeletal muscles, and conditions that limit glycogen synthesis in this tissue are associated with hyperglycemia and other metabolic disorders typically found in T2D patients^2^. Here, we show that Sol, EDL, and Epit muscles of HF rats had blunted insulin-stimulated glycogen synthesis and glucose oxidation rates, indicating that regardless of their fibre type distribution and capacity to oxidize fat, all muscles developed insulin resistance. Our original hypothesis was that, under conditions of chronic HF feeding, muscles such as Sol and EDL that are rich in type I and type IIA fibers would accumulate lower levels of DAG and ceramides in comparison to the Epit muscle rich in type IIB fibers. This would occur because more fatty acids would be oxidized in type I and IIB fibers leaving fewer to be diverted towards the formation of DAG and ceramides. This hypothesis was consistent with previous reports that PKCθ, considered the dominant isoform of DAG-sensing PKCs in skeletal muscle^25,26^, was more abundant in white than red rat muscles, and that insulin resistance was accompanied by DAG-mediated increase in PKCθ only in the membrane fraction of white muscles^16^. Indeed, we found that the total contents of PKCδ and PKCθ were much lower in Sol than in EDL and Epit muscles. However, analysis of the cytosolic and membrane fractions, revealed that the translocation of both PKCδ and PKCθ were markedly elevated in Sol muscles, whereas in EDL, only PKCδ translocation was increased by HF diet. Conversely, in Epit muscles, translocation of both PKC isoforms was reduced. These findings were consistent with the fact that HF feeding increased DAG and ceramides in Sol and EDL, but not in Epit muscles. Thus, contrary to our original hypothesis, high rates of fatty acid oxidation did not prevent DAG and ceramides accumulation and the induction of PKC translocation toward the membrane. Furthermore, our findings suggested that distinct mechanisms dictate insulin resistance under diet-induced obesity in red, white, and mixed muscles. In this context, we tested whether the expression of inflammatory mediators could also follow a distinct pattern among these muscles. Indeed, we found that even though circulating TNF-α and IL-6 were significantly increased in HF rats, only Epit muscles displayed an enhanced expression of major molecules that mediate inflammatory responses such as CD40 and FAS, TLR4, and NF-kB in these animals. TLR4 has been reported to selectively increase sphingolipid levels within the cell, suggesting that ceramides may be an important mediator of insulin resistance induced by TLR4 signalling. In fact, in order to induce insulin resistance in mice, the proinflammatory TLR4 has been shown to require the biosynthesis of ceramides^27^. Interestingly, in this study we found ceramides to be significantly elevated in Sol and EDL, but not in Epit muscles upon HF feeding. Thus, it appears that in Sol and EDL muscles the intracellular accumulation of DAG and ceramides contributed to the development of insulin resistance in these muscles, whereas in Epit muscles the signalling of inflammatory cytokines such as TNF-α and IL-6 through CD40 and FAS, TLR4, and NF-kB likely played a more relevant role in HF diet-induced insulin resistance.

Besides directly affecting insulin signalling and glucose metabolism in peripheral tissues, TNF-α and IL-6 have been shown to activate the hypothalamic–pituitary–adrenal axis (HPA) and stimulate adrenocorticotropic hormone (ACTH) and cortisol production^28^. Increased levels of glucocorticoids lead to dysfunctional alterations in glucose and lipid metabolism^19,29,30^. In fact, animal models with genetic predispositions to obesity and T2D such as the *ob/ob* mouse^31^ and the Zucker fatty rat^18^, as well as the HF diet-induced insulin resistance rat model^32^ show HPA hyperactivity. Additionally, treatment with dexamethasone has been reported to induce insulin resistance in oxidative and glycolytic rat skeletal muscles^29,30^, and severe hyperglycemia has also been recently described in HF-fed rats receiving exogenous corticosterone^33^. Conversely, adrenalectomy has been shown to reverse many of the metabolic abnormalities found in genetic models of obesity and insulin resistance^19,34^ and HF diet-induced insulin resistance in skeletal muscles was ameliorated in rats treated with an anti-glucocorticoid drug^20^. These previous observations are in line with our findings that skeletal muscle insulin resistance was accompanied by increased circulating corticosterone levels and also with upregulation of GR mRNA expression in EDL and Epit muscles in HF-fed rats. Furthermore, we found that the mRNA expression of 11β-HSD1, the enzyme that catalyses the intracellular conversion of circulating 11-dehydrocorticosterone into corticosterone in rodents^35^, was upregulated in Epit muscles from HF rats. Even though the mRNA expression of receptors for inflammatory cytokines, NF-kB, GR, and 11β-HSD1 were unaltered in Sol and EDL muscles of rats fed a HF diet, we cannot discard the possibility that elevated circulating TNF-α, IL-6, and corticosterone may have also contributed to some extent to the development of insulin resistance in these muscles.

Altogether, our findings provide evidence that the activation of PKCδ and PKCθ and pathways by which inflammatory cytokines and glucocorticoids signal in skeletal muscles are regulated by HF feeding in a muscle fiber type-specific manner. These findings could also explain why insulin resistance similarly develops in oxidative and glycolytic muscles of rats fed an obesogenic diet, despite major differences in the ability of these muscles to oxidize fat. Thus, the accumulation of DAG and ceramides appear to be important factors leading to PKC activation and disruption of insulin signalling and glucose metabolism in oxidative muscles under HF-fed conditions. However, because in highly glycolytic muscles DAG and ceramides were not significantly elevated and membrane-bound PKC was not altered, the activation of inflammatory pathways and upregulation of glucocorticoid signalling were more likely associated with HF diet-induced insulin resistance in these muscles.

## Materials and methods

### Reagents

Fatty acid (FA)-free bovine serum albumin (BSA), glycogen, and palmitic acid were obtained from Sigma (St. Louis, MO, USA). Diolein was from Nu-check (Elysian, MN, USA). Human insulin (Humulin R) was purchased from Eli Lilly Inc. (Toronto, ON, Canada). The insulin ELISA kit was from Millipore (Billerica, MA, USA). The Corticosterone RIA kit was from ALPCO Diagnostics (Salem, NH, USA). The TNF-α and IL-6 ELISA kits were from Invitrogen (Montreal, QC, Canada). The NEFA kit was from Wako Chemicals (Richmond, VA, USA). N‐Acetyl-D-sphingosine was from Sigma. The RNeasy Kit was from Qiagen Inc. (Toronto, ON, Canada) and the DNase from Thermo Fisher (Toronto, ON, Canada). D-[U-^14^C]glucose and [1-^14^C]palmitic acid were from GE Healthcare Radiochemicals (Quebec City, QC, Canada). The reverse phase column (C18 5 µm 250 × 4.6 mm) was from Restek (Bellefonte, PA, USA). The subcellular protein fractionation Kit was from Thermofisher (Cambridge, MA, USA). The PKCθ (cat # 13643), PKCδ (cat # 9616), Na/K ATPase (cat # 3010), and the GAPDH (cat # 2118) antibodies were from Cell Signalling Technology Inc. (Beverly, MA, USA).

### Animals

Male albino rats from the Wistar strain (Charles River Laboratories, Montreal, QC, Canada) weighing 200–250 g (initial weight) were maintained in a constant-temperature (23 °C), with a fixed 12-h light/12-h dark cycle and fed for 8 weeks ad libitum either a standard rat chow (Control, 27.0%, 13.0%, and 60.0% of calories provided by protein, fat, and carbohydrates, respectively, energy density 3.43 kcal/g) or a HF diet (20.0%, 60.0%, and 20.0% of calories provided by protein [casein], fat [lard/soybean oil], and carbohydrates [sucrose], respectively, energy density 5.24 kcal/g)^15^. The control diet (standard rat chow, catalog # 5012) was purchased from TestDiet (Richmond, IN, USA). The HF diet (catalog # D12492) was purchased from Research Diets Inc. (New Jersey, NJ, USA).

### Ethics approval

The protocol containing all animal procedures described in this study was specifically approved by the Committee on the Ethics of Animal Experiments of York University (York University Animal Care Committee, YUACC, permit number: 2021-03) and performed strictly in accordance with the YUACC guidelines. All tissue extraction procedures were performed under ketamine/xylazine anesthesia, and all efforts were made to minimize suffering^15^. All experiments in this study were carried out in compliance with the ARRIVE guidelines^36^.

### Determination of corticosterone, TNF-α, IL-6, and NEFAs in the serum

Blood from all animals was collected in the fed state between 09:00 and 10:00 by saphenous vein bleeding and the serum was used to determine plasma corticosterone, TNF-α, IL-6, and NEFAs using commercially available kits listed in the reagents section. All procedures were performed according to instructions provided by the manufacturers of the kits.

### Whole-body fat oxidation

At the end of the 8-week-diet-intervention period, all animals were placed in the comprehensive laboratory animal monitoring system (CLAMS) as previously described^37^. The CLAMS from Columbus Instruments (Columbus, OH, USA) perform automated in vivo determinations of oxygen consumption (VO_2_), carbon dioxide production (VCO_2_), and respiratory exchange ratio (RER). The animals were placed in the CLAMS at 12:00 and the first hour of data collected in the CLAMS was discarded, since it is the time required for the rats to fully acclimatize to the cage environment^37^. The rats were monitored for a 24 h period encompassing the light (07:00–19:00 h) and dark (19:00–07:00 h) cycles.

### Muscle isolation and incubation

All animals were anesthetized with a single intraperitoneal injection of ketamine/xylazine (90 mg and 10 mg/100 g B.W., respectively). Subsequently, Sol, EDL, and Epit muscles were quickly extracted. These muscles were chosen because of their wide range of reported fiber-type distributions with distinct mitochondrial contents and oxidative capacities. The percentages of type I, type IIa, and type IIb in Sol, EDL, and Epit muscles are 84/16/0, 3/57/40^38^, and 15/20/65^39^, respectively. Three sets of muscle strips (18–22 mg) were mounted onto thin stainless steel wire clips to maintain optimal resting length, and immediately placed in plastic scintillation vials containing 2 ml of pre-gassed [30 min with O_2_:CO_2_-95:5% (vol/vol)] Krebs–Ringer bicarbonate (KRB) buffer containing 4% fat-free BSA and 6 mM glucose. The vials were sealed with rubber stoppers and gasification was continued for the entire 1 h pre-incubation period. One set of muscles was then transferred to vials containing 2 ml of the same KRB buffer plus D-[U-^14^C]glucose (0.2 µCi/ml) and incubated under continuous gasification for one additional hour either in the absence (basal) or presence of insulin (100 nM) for the determination of glycogen synthesis^40^. For the assessment of glucose oxidation, a centered isolated well containing a loosely folded piece of filter paper moistened with 0.2 ml of 2-phenylethylamine/methanol (1:1, vol/vol) was inserted into the flasks where the muscles were incubated. After the 1 h incubation period, the muscles were removed, and the media were acidified with 0.2 ml of H_2_SO_4_ (5 N). The flasks were maintained sealed at 37 °C for an additional 1 h for collection of the ^14^CO_2_ released. Subsequently, the filter papers were carefully removed and transferred to scintillation vials for radioactivity counting.

### Measurement of glycogen synthesis in isolated muscles

Glycogen synthesis was assessed by measuring the incorporation of D-[U-^14^C]glucose into glycogen as previously described^40^. Briefly, immediately after incubation, muscle strips were quickly washed in ice-cold PBS, blotted on filter paper, frozen (N_2_), and digested in 0.5 ml of KOH 1 M at 70 °C for 1 h. Of the digested muscle solution, aliquots were taken for the determination of glycogen synthesis. Glycogen was precipitated overnight (− 20 °C) with 100% ethanol, resuspended in 0.5 ml of water, and its radioactivity was determined using a scintillation counter.

### Measurement of palmitate oxidation in isolated muscles

Palmitate oxidation was measured by assessing the production of ^14^CO_2_ from [1-^14^C]palmitic acid. The flasks where muscle strips were incubated contained 2 ml of KRB buffer plus 0.2 mM of cold palmitic acid previously complexed with FA free BSA and [1-^14^C]palmitic acid (0.2 µCi/ml). The muscles were incubated under continuous gasification for 1 h and the vials had a centered isolated well containing a loosely folded piece of filter paper moistened with 0.2 ml of 2-phenylethylamine/methanol (1:1, vol/vol). After the 1 h-incubation period, the muscles were quickly removed and the media were acidified with 0.2 ml of H_2_SO_4_ (5 N), and the flasks were maintained sealed at 37 °C for an additional 1 h for collection of the ^14^CO_2_ released. Subsequently, the filter papers were carefully removed and transferred to scintillation vials for radioactivity counting^41^.

### Determination of DAG and ceramides contents in Sol, EDL, and Epit muscles

The ultra-high-pressure liquid chromatography system (UHPLC-UV, Nexera X2, Shimadzu, Kyoto, Japan) was used to measure total amounts of DAG in lipid samples extracted from Sol, EDL, and Epit muscles using the Folch’s method^42^. Briefly, 150 mg of muscle tissue were homogenized in 200 µl of chloroform:methanol (MeOH) [2:1 vol/vol], dried overnight under nitrogen gas, and resuspended in 100 µl of 2-propanol-hexane (ProHex, 5:4 vol/vol) prior to chromatographic analysis. Quantification was performed using the UHPLC-UV detection machine. Sample volumes (50 μl) were injected automatically into a reverse phase column (C18 5 µm 250 × 4.6 mm). The chromatography conditions were set to 40 °C for 20 min using a gradient of MeOH and ProHex:100% of MeOH from 0 to 10 min, followed by 50% of MeOH and 50% of ProHex for 10 min, maintained with isocratic elution for 10 min. Diolein (0.25 µg/µl) were also dissolved in ProHex and quantified to obtain a standard curve^43^. To analyse total ceramide content, a small volume of the lipid extract obtained after chloroform extraction was transferred into new pre-weighed eppendorfs as previously described^44^. The organic phase was hydrolyzed in 1 M KOH at 90 °C for 60 min. The sphingosine liberated from ceramides was analyzed by means of UHPLC by mixing it with 15 µl OPA reagent and allowing it to derivatize for 20 min at room temperature. The calibration curve was prepared using N‐Acetyl-D-sphingosine as a standard. The samples were reconstituted in 100 μl of chloroform–methanol-acetic acid–water (50:37.5:3.5:2 vol/vol/vol/vol) and run through a porous silica column (ARC-18 1.8 µm 100 × 2.1 mm). Elution was conducted with heptane-isopropyl ether-acetic acid (60:40:3 vol/vol/vol) at a gradient from 0 to 10% in 30 min at a flow rate of 0.8 ml/min followed by isocratic elution with acetonitrile: deionized distilled water (90:10, vol/vol) and a flow rate of 1 ml/min^45^. Subsequently, the column was equilibrated with chloroform–methanol-acetic acid–water (50:37.5:3.5:2 vol/vol/vol/vol) for 10 min at the same flow rate.

### Tissue fractionation and Western blotting analysis of PKCδ and PKCθ content and cellular localization in Sol, EDL, and Epit muscles

PKCδ and PKCθ protein levels were determined in the cytosolic and membrane fractions by immunoblotting with each respective PKC-specific antibody. Muscle homogenates (50 mg) were used to attain the cytoplasmic and membrane protein fractions using a fractionation kit. The separated fractions were then collected, and respective aliquots were used to measure the protein content by the Bradford method. Samples were then diluted 1:1 (vol/vol) with 2 × Laemmli sample buffer, heated to 95 °C for 5 min, and subjected to SDS-PAGE. PKCδ and PKCθ-specific antibodies (1:2,000 dilution) were used determine the subcellular localization of these proteins. Normalization of the cytoplasmic and membrane fractions of PKCδ and PKCθ was performed by first dividing the cytoplasmic and membrane densitometric values by GAPDH and Na/K ATPase, respectively. Subsequently, the normalized values for the membrane and cytoplasmic fractions were divided by each other to obtain the membrane/cytoplasmic (mem/cyto) ratios that are expressed as arbitrary units (AU) in Fig. 6. We were able to probe for PKCδ and PKCθ membrane fractions and Na/K ATPase using the same membrane. Therefore, the same Na/K ATPase blot for each muscle could be used to normalize the membrane fractions of both PKC isoforms. This is the reason why the same Na/K ATPase representative blot is shown for each individual muscle in Fig. 6. However, because we ran two separate membranes for PKCδ and PKCθ cytoplasmic fractions, separate membranes were also used to probe for GAPDH and normalize the cytoplasmic PKCδ and PKCθ fractions. This is why we show in Fig. 6 different GAPDH representative blots for cytoplasmic PKCδ and PKCθ fractions. Full PKC blots can be found in supplementary information.

### Quantitative PCR analysis

Total RNA was isolated from skeletal muscles using the RNeasy kit, followed by DNase treatment in order to remove genomic DNA carry-over. Primers were designed using the software PrimerQuest (IDT) based on probe sequences available at the Affymetrix database (NetAffx Analysis Center, http://www.affymetrix.com/analysis) for each given gene. Real-time PCR reactions were carried out at amplification conditions as follows: 95 °C (3 min); 40 cycles of 95 °C (10 s), 65 °C (15 s), 72 °C (20 s); 95 °C (15 s), 60 °C (15 s), 95 °C (15 s). Quantitative PCR was performed using the CFX96 Real-time system from Bio-Rad. All genes were normalized to the control gene TBP, and values are expressed as fold increases relative to control. Primers sequences utilized are shown in Table 2.

**Table 2:** Primers used for qPCR.

Gene	Forward	Reverse
FAS	TGGCTGTGTTCTGGACTTAAA	GTATCCCTGCTCATGATGTCTAC
CD40	AGATTATCCCGGTCACAACAC	CTGAGATGCGACTCTCTTTACC
TLR4	ACCTAAGGAGAGGAGGCTAAG	GGTAACTGCAGCACACTACA
NF-kB	TCCAGCTGCTATTGGATTACAC	GGGACTGCGATACCTTAATGAC
GR	GAAGGGAACTCCAGTCAGAAC	AATGTCTGGAAGCAGTAGGTAAG
11β-HSD1	CTCCACTTCTGCTTGGGAAT	CTCAGGAGTTCCTAGTTGCTTAC
TBP	TACAGGTGGCAGCATGAAGTGACA	AACCAACAATCACCAGCAGCAGTG

### Statistical analyses

Data were expressed as Mean ± SE. Statistical analyses were performed by using Two-way ANOVA with Tukey–Kramer multiple comparison post-hoc test or *t*-tests as indicated in the figure legends. Parametric tests selected for statistical significance were based on normality tests performed on the data. The GraphPad Prism software version 9.1.12 was used for all statistical analyses and for the preparation of all graphs. The level of significance was set to p < 0.05.

## Supplementary Information

Supplementary Information.

## Data Availability

The datasets generated during and/or analyzed during the current study are available from the corresponding author on reasonable request.
