# Supplementary material for: Distinct mechanisms involving diacylglycerol, ceramides, and inflammation underlie insulin resistance in oxidative and glycolytic muscles from high fat-fed rats
Source: Sci Rep. 2021 Sep 27;11:19160. doi: 10.1038/s41598-021-98819-7 (PMC8476522; doi:10.1038/s41598-021-98819-7)
Supplement: Supplementary file 1 — Supplementary Information. [file 41598_2021_98819_MOESM1_ESM.pdf]

Distinct mechanisms involving diacylglycerol, ceramides, and inflammation underlie insulin resistance in oxidative and glycolytic muscles from high fat-fed rats

Shailee Jani[1], Daniel Da Eira[1], Ishvinder Hadday[1], George Bikopoulos[1], Arta Mohasses[1], Ricardo A. de Pinho[2], and Rolando B. Ceddia[1]

[1]Muscle Health Research Center – School of Kinesiology and Health Science, York University, North York, ON, Canada. [2]Graduate Program in Health Sciences, School of Medicine, Pontifícia Universidade Católica Do Paraná, Curitiba, Paraná, Brazil.

Supplementary data: Full length blots

Total PKCδ and β-actin

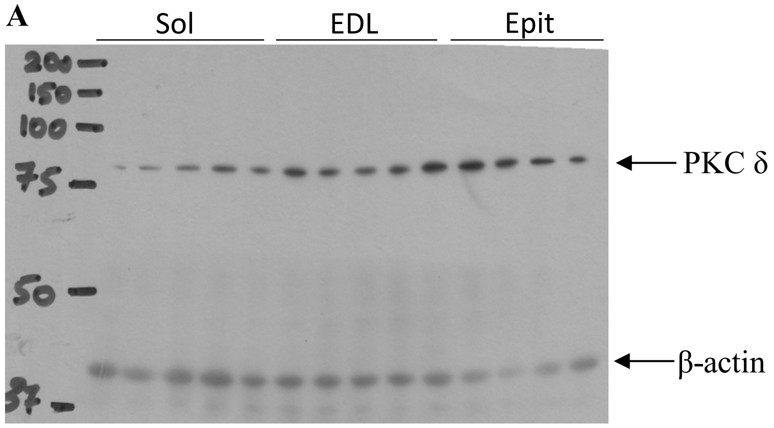

Total PKCθ

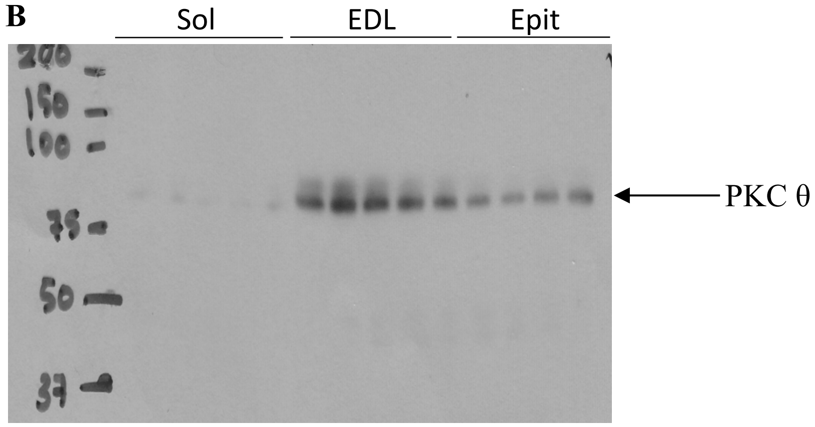

PKCθ βactin

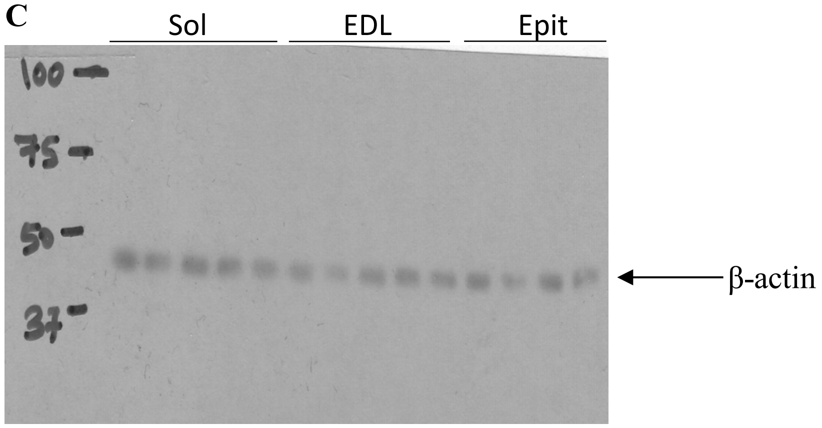

Supplementary Figure 1. Full blots showing total PKCδ and β-actin (A) and PKCθ (B) and β-actin (C) in Sol, EDL and Epit muscles.

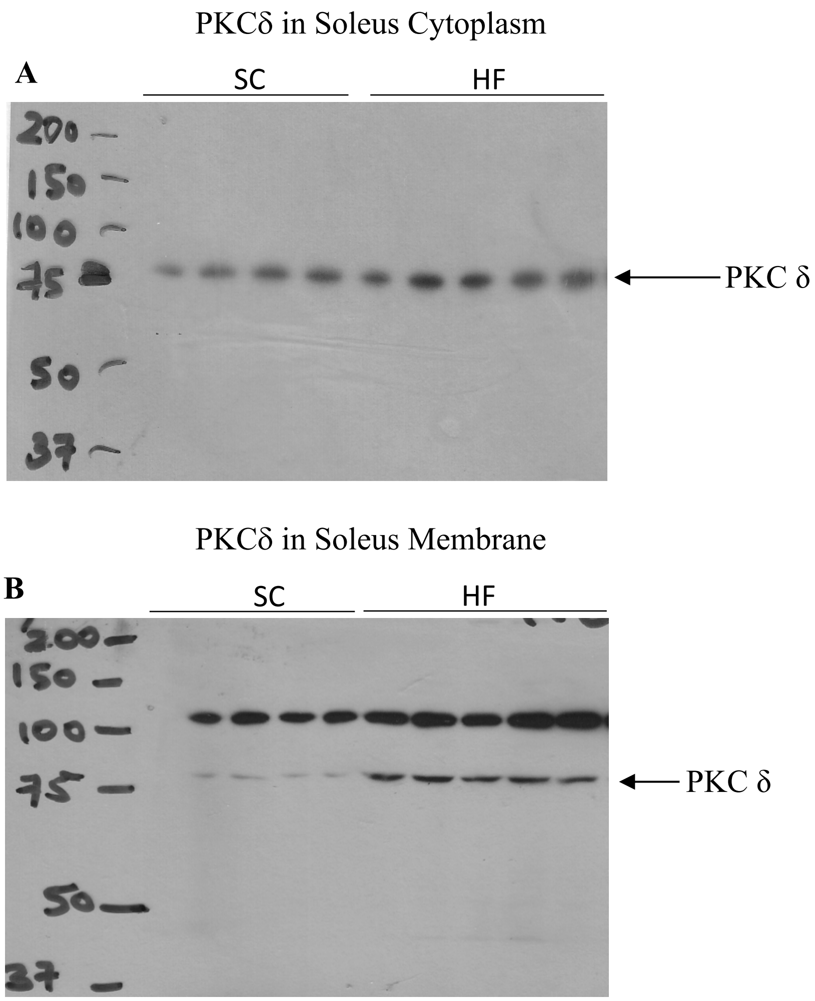

Supplementary Figure 2. Full blots showing cytoplasmic PKCδ (A) and membrane-bound PKCδ (B) in Sol muscles of SC and HF-fed rats.

PKCδ in EDL Cytoplasm

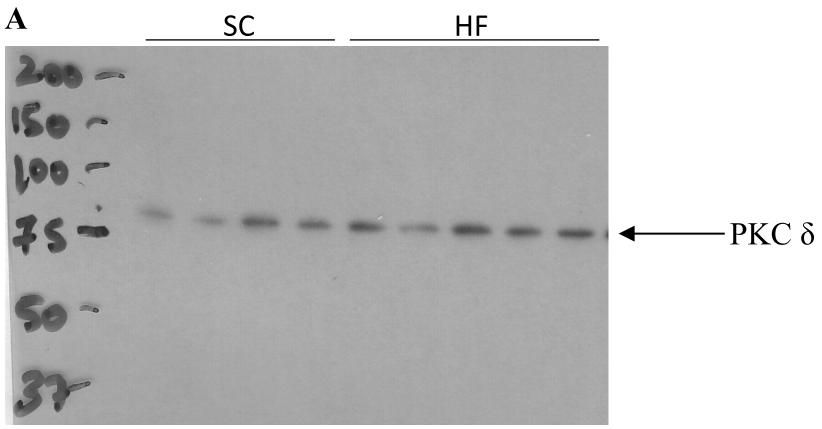

PKCδ in EDL Membrane

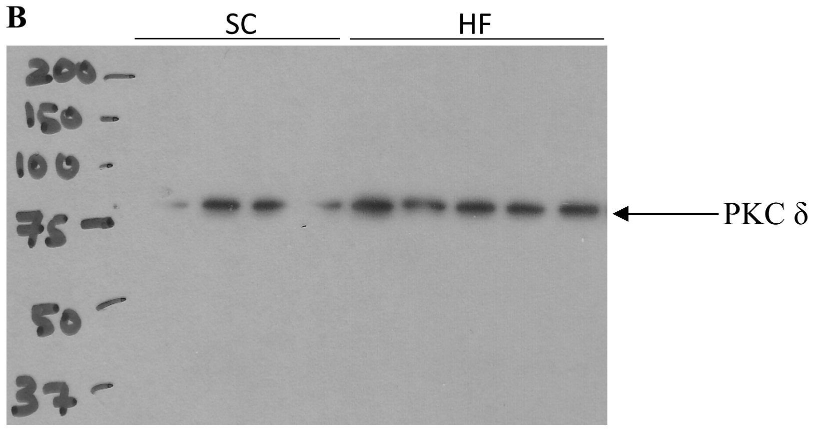

Supplementary Figure 3. Full blots showing cytoplasmic PKCδ (A) and membrane-bound PKCδ (B) in EDL muscles of SC and HF-fed rats.

PKCδ in Epit Cytoplasm

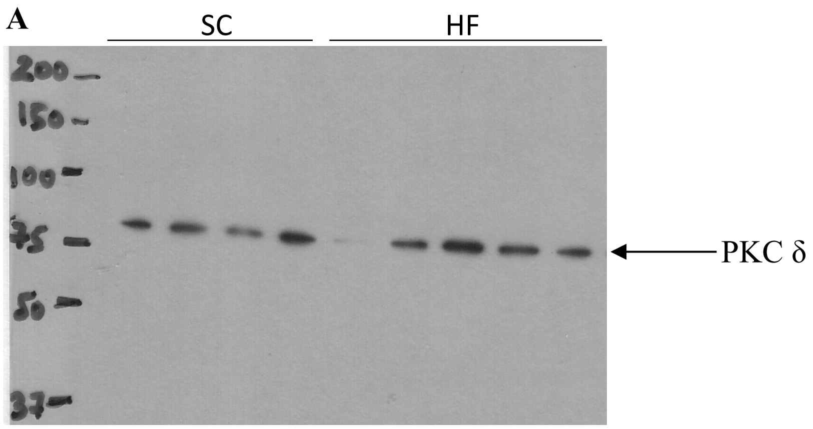

PKCδ in Epit Membrane

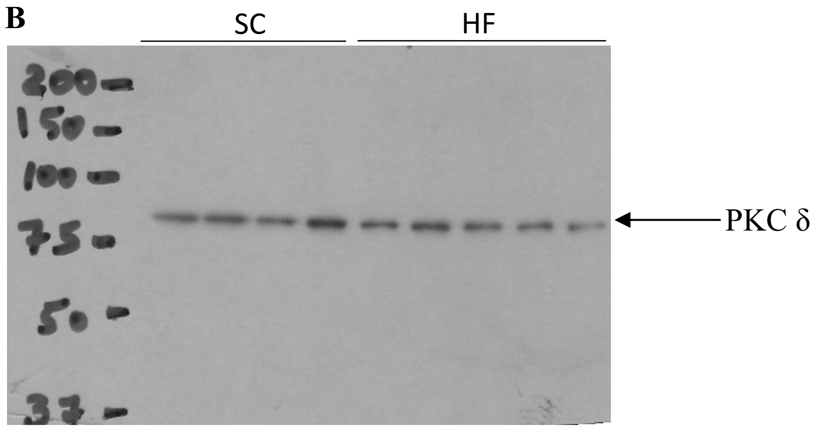

Supplementary Figure 4. Full blots showing cytoplasmic PKCδ (A) and membrane-bound PKCδ (B) in Epit muscles of SC and HF-fed rats.

PKCθ in Soleus Cytoplasm

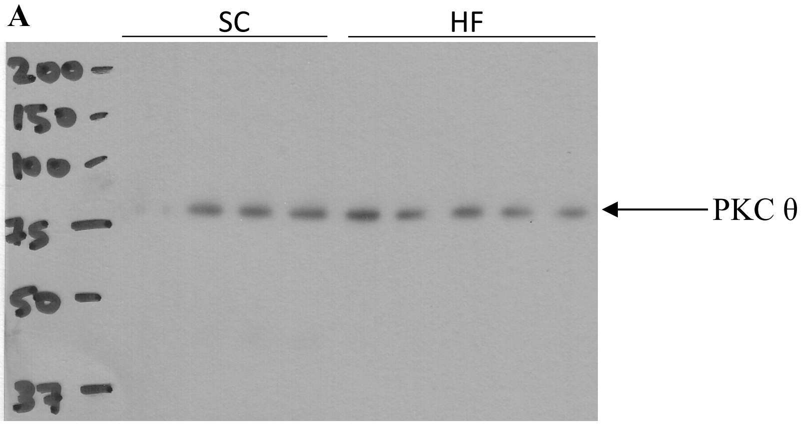

PKCθ in Soleus Membrane

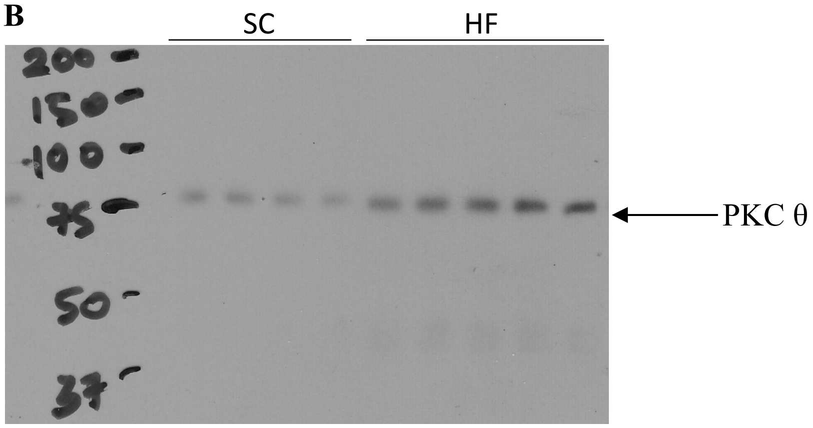

Supplementary Figure 5. Full blots showing cytoplasmic PKCθ (A) and membrane-bound PKCθ (B) in Sol muscles of SC and HF-fed rats.

PKCθ in EDL Cytoplasm

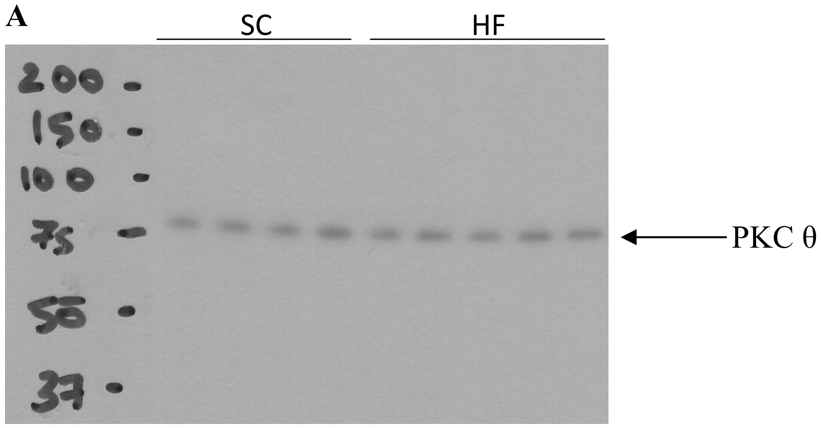

PKCθ in EDL Membrane

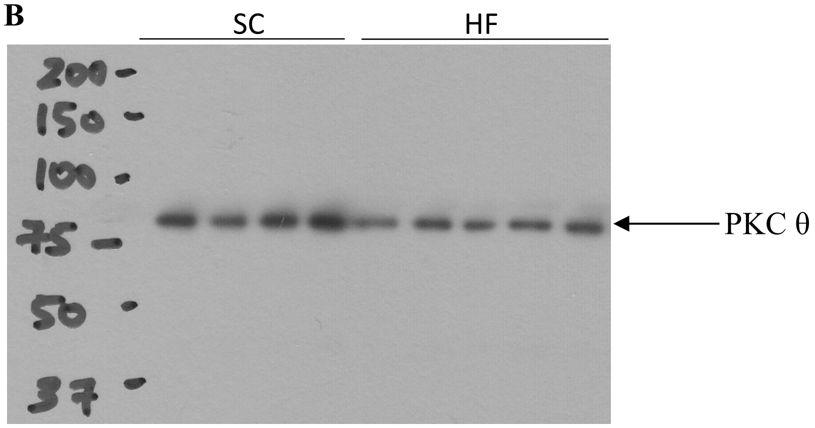

Supplementary Figure 6. Full blots showing cytoplasmic PKCθ (A) and membrane-bound PKCθ (B) in EDL muscles of SC and HF-fed rats.

PKCθ in Epit Cytoplasm

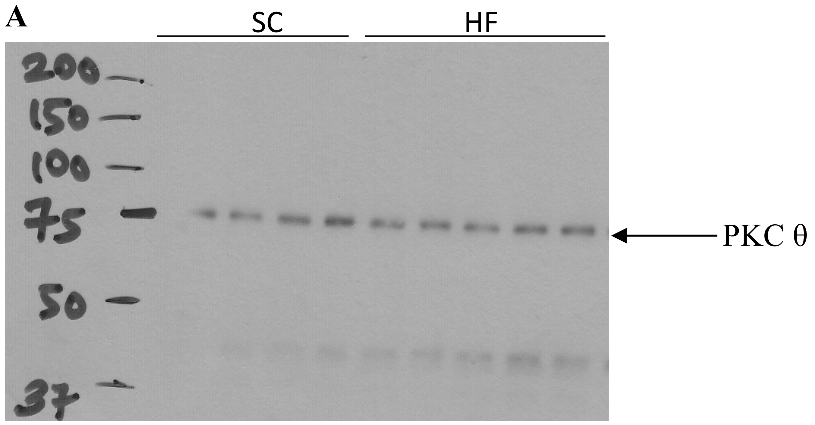

PKCθ in Epit Membrane

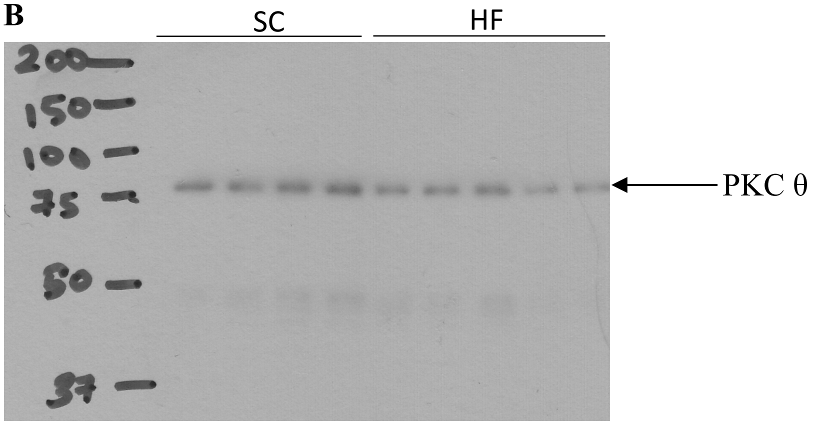

Supplementary Figure 7. Full blots showing cytoplasmic PKCθ (A) and membrane-bound PKCθ (B) in Epit muscles of SC and HF-fed rats.
